# Evaluation of Microarray Preprocessing Algorithms Based on Concordance with RT-PCR in Clinical Samples

**DOI:** 10.1371/journal.pone.0005645

**Published:** 2009-05-21

**Authors:** Balazs Gyorffy, Bela Molnar, Hermann Lage, Zoltan Szallasi, Aron C. Eklund

**Affiliations:** 1 Hungarian Academy of Sciences and Semmelweis University Budapest, Budapest, Hungary; 2 Charité Universitätsmedizin Berlin, Institute of Pathology, Berlin, Germany; 3 Center for Biological Sequence Analysis, Technical University of Denmark, Lyngby, Denmark; 4 Children's Hospital Boston, Boston, Massachusetts, United States of America; Baylor College of Medicine, United States of America

## Abstract

**Background:**

Several preprocessing algorithms for Affymetrix gene expression microarrays have been developed, and their performance on spike-in data sets has been evaluated previously. However, a comprehensive comparison of preprocessing algorithms on samples taken under research conditions has not been performed.

**Methodology/Principal Findings:**

We used TaqMan RT-PCR arrays as a reference to evaluate the accuracy of expression values from Affymetrix microarrays in two experimental data sets: one comprising 84 genes in 36 colon biopsies, and the other comprising 75 genes in 29 cancer cell lines. We evaluated consistency using the Pearson correlation between measurements obtained on the two platforms. Also, we introduce the log-ratio discrepancy as a more relevant measure of discordance between gene expression platforms. Of nine preprocessing algorithms tested, PLIER+16 produced expression values that were most consistent with RT-PCR measurements, although the difference in performance between most of the algorithms was not statistically significant.

**Conclusions/Significance:**

Our results support the choice of PLIER+16 for the preprocessing of clinical Affymetrix microarray data. However, other algorithms performed similarly and are probably also good choices.

## Introduction

Preprocessing of raw probe intensities is an essential procedure in the analysis of gene expression microarray data. Generally, background correction and normalization are used to reduce the impact of variations in experimental conditions. Arrays featuring multiple probes per gene target (such as those from Affymetrix) require an additional probe-level summarization step to integrate intensities of the multiple probes into a single expression estimate. These steps involving background correction, normalization, and summarization are often combined into a single all-in-one preprocessing algorithm that takes raw probe intensities as input and produces gene expression estimates as output. Several such algorithms have been proposed, each based on a different model or set of underlying assumptions. Because the choice of algorithm can affect the conclusions drawn from the data, it is important to compare the accuracy of these algorithms.

The performance of preprocessing algorithms has been characterized in several ways, often with the help of data sets created expressly for this purpose (*evaluative data sets*). For example, the Latin square spike-in data sets from Affymetrix are derived from composite RNA samples, in which a small number of exogenous RNA species are added at various concentrations to a fixed background. Here, the known concentrations of the exogenous genes can be used as a reference to evaluate the accuracy of the microarray expression values [1], [2]. In another approach, mixture experiments involve two or more biological samples combined in various ratios, such that many genes are differentially expressed and the linearity of response can be quantified [3]. Such data sets have been the basis for systematic comparison of preprocessing methods [4], [5]. However, these data sets were generated under well-controlled conditions with relatively little variation in experimental or technical conditions from sample to sample. In this way, these data are not representative of typical microarray experiments performed in a research setting, such as those involving clinical samples [6], [7].

Therefore, it is also important to evaluate the performance of preprocessing algorithms on microarray data created to address biological or clinical research questions (*investigational data sets*) [7], [8]. In such data, there are no gold standards to use to directly assess accuracy. Instead, the relative performance of preprocessing algorithms can be assessed using independent measurements, such as a second microarray platform or quantitative real-time PCR (RT-PCR) [9], [10]. Unfortunately, only a few algorithms have been compared in this manner, and the data is not publicly available.

Here, we aimed to compare Affymetrix preprocessing algorithms using data sets that are representative of our research. We selected two investigational data sets, each featuring a relatively large number of matched measurements by Affymetrix microarrays and RT-PCR, and used these data sets to compare the accuracy of nine published preprocessing algorithms.

## Results

### Two representative investigational data sets

We chose to analyze two of our recently published data sets in which RNA samples had been profiled both on gene expression microarrays (Affymetrix) and on 96-well RT-PCR arrays (TaqMan). The first data set comprises 36 colon biopsies [11], and the second comprises 29 cancer cell lines grown *in vitro* [12]. All data was generated for previously published studies, without consideration for its present use in this study of preprocessing algorithms. Using current manufacturer-supplied annotations, we selected 84 probe sets in the colon cancer data set and 75 probe sets in the cell line data set for which we had comparable RT-PCR measurements (i.e. detecting the same set of transcripts). Evaluation of consistency between the two platforms was restricted to these matched measurements, which we call “genes” even though some splice isoforms may not be detected.

**Figure 1:**
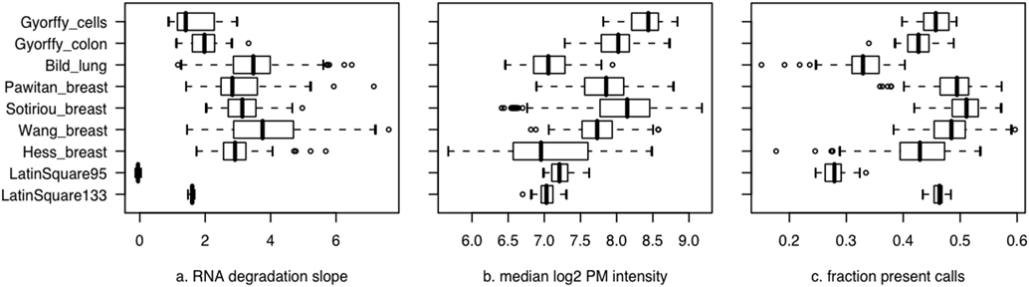
The colon and cell line data sets are representative of clinical microarray data. For several Affymetrix data sets, box-and-whiskers plots indicate the distribution of three bias metrics: a) RNA degradation slope, b) median perfect-match probe intensity, and c) fraction of probe sets called present. A narrower distribution indicates greater consistency in technical conditions. *LatinSquare133* and *LatinSquare95* are spike-in data sets produced by the microarray manufacturer [1]; *Gyorffy_cells* and *Gyorffy_colon* are the data sets analyzed in this paper [11], [12]; the other five are publicly-available clinical data sets [14]–[18].

To assess whether these two data sets were representative of typical clinical data sets, we utilized three “bias metrics” as indicators of technical variation [13]. These bias metrics, which are calculated from the raw probe intensities, correspond to technical aspects of the experiment, such as hybridization conditions or RNA quality. In general, the expression values of many genes are correlated with these bias metrics [13]. Thus, the variation in bias metrics is an estimate of one type of noise that we expected to be higher in clinical data sets than in spike-in data sets. We compared the variation in bias metrics among several data sets: the two data sets analyzed in this paper, two Latin square spike-in data sets, and five tumor data sets that are relevant to our research interests [14]–[18]. We found that the colon and cell line data sets analyzed in this study are comparable to the tumor data sets, whereas the spike-in data sets exhibited much lower variation in the bias metrics (Figure 1). Thus, by this measure, the two data sets analyzed in this study are representative of “typical” clinical data sets, whereas the spike-in data sets are not.

### Preprocessing algorithms

Several preprocessing algorithms for Affymetrix-type oligonucleotide arrays have been proposed, but we evaluated only algorithms that were published or referenced in peer-reviewed journals and that were implemented in the R statistical environment. We identified the following nine algorithms meeting these criteria: DFW [19], FARMS [20], GCRMA [21], MAS5 [22], MBEI [23], PLIER [24], RMA [25], and VSN [26]. Two of these algorithms, MBEI and PLIER, were not implemented in R by the original authors, so we used published implementations of these algorithms [27], [28]. Although all of these algorithms have adjustable parameters, we generally used the default parameter values. As an exception, alternative parameter values for PLIER were chosen by the manufacturer for use in a large evaluative study, so we evaluated these settings (PLIER+16) in addition to the defaults [3]. By evaluating only previously published parameter values, we avoided problems of selection bias or overfitting that would result if we had tested additional parameter values.

### Evaluation of consistency between platforms

To a first approximation, the measured probe intensity is proportional to its target transcript concentration, but is also affected by other factors such as probe sequence. Therefore, the absolute expression of a single gene in a single sample is not directly comparable between different gene expression platforms. However, the relative expression of a gene between two samples should be directly comparable, as long as the normalization factor is comparable. Microarray data is often normalized globally, meaning that the distribution of expression levels of all genes is assumed to be constant in all samples. On the other hand, RT-PCR data is often normalized using a small number (e.g., one) of housekeeping genes that are assumed to be constant. For our study, to make the normalization of the microarray and RT-PCR data as similar as possible, we normalized each sample according to the median expression value of the common genes. This additional normalization step increased concordance in both data sets (data not shown).

**Figure 2:**
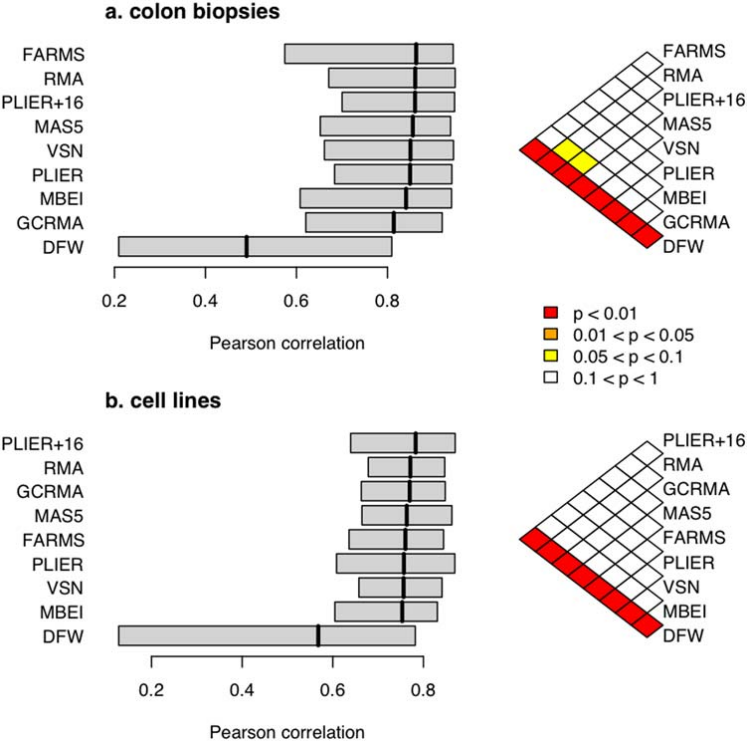
Pearson correlation coefficients between microarray and RT-PCR. The distribution of Pearson correlation coefficients for each microarray preprocessing algorithm is indicated by a box plot, for a) the colon cancer data set (84 genes, 36 samples), and b) the cell line data set (75 genes, 29 samples). The box indicates the 25th to 75th percentile, and the heavier line indicates the median. Algorithms are displayed in decreasing order of the median, such that the more accurate algorithms are at the top. The colorgrams on the right-hand side indicate *P* values (Wilcoxon test) comparing each pair of algorithms.

To assess concordance between the two platforms, we treated each gene as an independent measurement. For each gene, we calculated the Pearson correlation coefficient (PCC) between the log_2_-transformed expression values as measured by microarray and those measured by RT-PCR. We then compared the distributions of PCCs obtained using the various preprocessing algorithms (Figure 2). Using the median of the PCC distribution as the criterion to choose the most accurate algorithm, FARMS was the most accurate on the colon data set, while PLIER+16 was most accurate in the cell line data set. In both data sets, the difference between the majority of the algorithms was not statistically significant (Figure 2).

However, the use of PCC as measurement of concordance can be problematic for two important reasons. First, a gene that is essentially unchanging often has low PCC due to random noise, even if both platforms agree that the gene is unchanging. Second, a gene can have high PCC even if the two platforms are highly discordant in magnitude; for example, when one platform experiences compression due to chemical saturation at higher intensities.

We sought to define a second metric for cross-platform comparison that more intuitively indicates the agreement or disagreement between sets of measurements. In practice, we are most interested in the extent to which a gene measured on the two platforms has the same log ratio for a given pair of samples. Thus, for each gene *g* we define the log-ratio discrepancy (LRD) as:
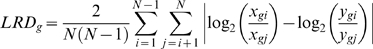
where *N* is the number of samples, and *xgs* and *ygs* are the expression values of gene *g* in sample *s*, as measured on each platform. In simpler terms, the LRD of a gene is the absolute difference in log-ratio as measured between platforms, averaged over all possible pairs of samples. Therefore, the LRD has a minimum possible value of zero (perfect agreement) and a maximum value limited only by the dynamic range of the two platforms. An R script implementing the LRD is available in Text S1.

**Figure 3:**
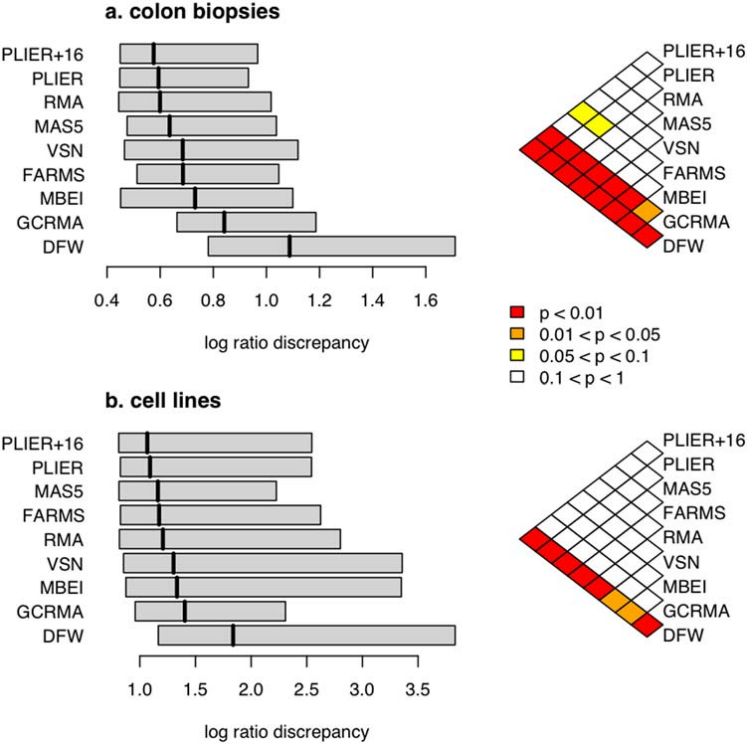
Log-ratio discrepancy between microarray and RT-PCR. The distribution of the log-ratio discrepancy for each microarray preprocessing algorithm is indicated by a box plot, for a) the colon cancer data set, and b) the cell line data set. Algorithms are displayed in order of the median, such that the more accurate algorithms are at the top. The colorgrams on the right-hand side indicate *P* values (Wilcoxon test) comparing each pair of algorithms.

We calculated the LRD between the microarray and RT-PCR expression values for each gene and compared the LRD distribution between the nine preprocessing algorithms (Figure 3). Using the median of the LRD values as the criterion to choose the best performing preprocessing algorithm, we found PLIER+16 at the top of the list in both data sets. Again, several other algorithms showed similar performance, and the difference between the top few algorithms was not statistically significant (Figure 3).

## Discussion

Several preprocessing algorithms have been proposed for Affymetrix arrays, but so far it is unknown whether one particular algorithm provides more accurate results. Several important studies attempting to answer this question using artificially produced RNA samples have been a key source of guidance for investigators [2], [4]. Until now, there has been a lack of systematic comparisons of the performance of preprocessing algorithms in investigational studies. In this study, we have evaluated nine preprocessing algorithms in two complementary data sets that are representative of typical microarray experiments that we generate and analyze in our research. Because none of the true gene expression levels are known exactly, we assessed the accuracy of the various preprocessing algorithms by comparing the generated expression values with those measured independently on RT-PCR arrays.

RT-PCR is often used to “confirm” microarray results because of its relatively accurate measurements over a wide dynamic range. However, the interpretation of these results can be problematic because RT-PCR expression values can vary based on choice of normalization controls [29]. Although our study has been designed in a way to avoid standard housekeeping gene-based normalization of RT-PCR data, this task is important for accurate results in actual research situations. Despite the fact that no single gene is expressed at a constant level in all biological samples, RT-PCR measurements are often normalized to a single gene. Several publications have introduced more rational normalization methods for RT-PCR. A model-based variance estimation approach was introduced to identify genes with the lowest variance in a given type of data set and therefore best suited for normalization [30]. In another approach, the geometric average of multiple control genes was found to be an accurate normalization factor for RT-PCR measurements [29].

Although the accuracy of preprocessing algorithms for Affymetrix microarrays could be compared in numerous ways, we have used two performance metrics. First, we used the PCC because it is intuitive and because it gives a useful measure of concordance under many conditions. Additionally, we introduced the log-ratio discrepancy, which we believe is a more useful assessment of expression value accuracy in the context of our biomarker research due to its ability to take into consideration the impact of genes that do not change their expression across the various samples in the cohort. Researchers using microarrays for other types of experiments may find other performance metrics to be more relevant to their research, and our results should be considered in this context. For example, the LRD penalizes compression-type artifacts; whereas PCC does not. Such artifacts may be acceptable for simple analysis such as searching for differentially expressed genes between two relatively homogeneous groups. However, such artifacts are not acceptable when the response must be linear, e.g. in principal components analysis. An open question is whether the optimal choice of preprocessing algorithm is dependent on the type of data, or on the biological question being asked. For example, some analysis, such as regulatory network reconstruction, may be highly sensitive to random correlations, in which case concordance with RT-PCR measurements may not be the only consideration for selecting an algorithm [13], [31].

Naturally, our choice of data sets from colon biopsies and cancer cell lines reflects our research interests. These data sets, like many recent large-scale data sets, were generated using the HG-U133A or HG-U133 Plus 2.0 microarrays, which use a 3′-biased amplification protocol and a transcript-based probe set design. In contrast, the newer generation of microarrays from Affymetrix utilizes a whole-transcript amplification protocol and an exon-based probe design, which may offer a more specific portrait of expressed sequences [32]. Preprocessing algorithms that perform well in the U133 arrays should also perform well in the newer arrays, but it will be important to confirm this as researchers adopt the newer platforms.

Several other studies have compared preprocessing algorithms using different data sets and/or different metrics. Cope *et al.* described a series of tests to evaluate preprocessing algorithms using the spike-in data set from Affymetrix and the dilution data set from Genelogic. They generally observed better performance from RMA and MAS5 than from dChip (MBEI), which is consistent with our findings. Choe *et al.* generated a spike-in data set with a defined background, in contrast to the Affymetrix spike-in data sets, which use HeLa RNA as background [2]. Instead of comparing all-in-one preprocessing algorithms as we did, they evaluated the relative merit of the individual background correction, normalization, and summarization steps. Dallas *et al.* compared Affymetrix arrays with RT-PCR measurements of 48 genes in various human samples using PCC as the performance metric [8]. They evaluated only MAS5 and RMA and found that the two algorithms had comparable performance. Qin *et al.* compared microarrays to RT-PCR using mouse heart tissue and Pearson correlation of fold change as a performance metric, and found that MAS5, dChip-with-mismatch, and GCRMA outperformed dChip-without-mismatch (analogous to our “MBEI”), RMA, and VSN [7]. Barash *et al.* used variability in redundant measurements to estimate noise, and found that RMA outperformed dChip and MAS5 without decreasing the number of differentially expressed genes [6]. There is some disagreement between these results and ours, which might stem from differences in data sets and methodology.

It is our impression that the most commonly used algorithms are MAS5, MBEI, and RMA [33]. This may be partially due to their relatively early availability (2001–2003) or partially due to their packaging within relatively user-friendly software (MAS5 and MBEI) or fast computation in R (RMA). Therefore, it is reassuring that these three algorithms performed well in our tests, although MBEI was slightly weaker. We were somewhat surprised by the performance of the MAS5 algorithm, which has not always performed well in earlier comparisons [21], [25]. Notably, MAS5 is the only algorithm in our comparison that operates on a single array at a time, a feature that is advantageous for diagnostic use but may represent a handicap compared to the other algorithms, which theoretically gain accuracy by considering an entire series of arrays in a single statistical model. The good performance of the MAS5 algorithm has been noted by others [34].

Overall, if we had to choose a single preprocessing algorithm, we would choose PLIER+16. However, several other algorithms performed in a comparable manner. Ultimately, it is likely that any of the top algorithms would be suitable for most purposes.

## Materials and Methods

### Samples

36 colon biopsies were taken during routine endoscopical intervention before treatment at the 2nd Department of Internal Medicine at the Semmelweis University Budapest and have been described previously [11]. The biopsies were classified as adenoma with (n = 6) or without dysplasia (n = 6), ulcerative colitis (n = 7), Crohn's disease (n = 1), colon cancer Dukes B (n = 6), colon cancer Dukes C-D (n = 5) and normal tissue (n = 5). 29 human cancer cell lines representing various cancer types were cultured under identical conditions and collected during logarithmic growth phase and have been described previously [12].

At the beginning of sample collection the written informed consent of patients was obtained for the use of their samples for diagnostic and scientific purposes. At the beginning of data processing all samples received a unique three digit number, and during further analyses this number was used without referring to the patient's name. The ethical committee of the Semmelweis University approved the study; the study conformed to the principles outlined in the Declaration of Helsinki.

### Measurements

Samples were profiled using HG-U133A or HG-U133 Plus 2.0 microarrays (Affymetrix, Santa Clara), and RT-PCR measurements were made on TaqMan 96-well arrays (Applied Biosystems, Foster City) as described [12]. Some of the genes on the TaqMan arrays were selected to validate our previous microarray studies and have been published earlier [12], while others were selected based on published clinical associations (e.g. colon cancer associated genes) or as normalization controls. One sample from the cell line data set was identified as low-quality (based on a very low number of genes detected as present on the microarray) and was removed from further analysis. Nonetheless, we have made the raw, unfiltered data sets publicly available in their entirety (GEO accession numbers GSE4183 and GSE11812 for microarrays; Dataset S1 and Dataset S2 for RT-PCR).

### Identification of common genes

We identified the targets (gene symbol and Refseq accession numbers) of the RT-PCR probes using online annotation provided by the manufacturer (accessed May 23, 2008). We identified the targets of the microarray probe sets using annotation files downloaded from the manufacturer's website (dated March 18, 2008). To find comparable sets of measurements made on both platforms, we identified RT-PCR probes and microarray probe sets that queried not only the same gene, but also the same set of transcript isoforms as determined by Refseq accession numbers. For several genes, multiple microarray probe sets matched an RT-PCR probe; in this case we chose the probe set with the most individual probes matching the queried gene, and if this criterion was not sufficient to choose a single probe set, we arbitrarily chose the first probe set from those matching the criteria. By this procedure we identified 84 genes in the colon cancer data set and 75 genes in the cell line data set measured by both platforms (Table S1). Only these genes were used to assess agreement between platforms.

### Data analysis

All analysis was performed using the R statistical environment (www.R-project.org) with Bioconductor packages (www.bioconductor.org). All calculations and comparisons were performed using log_2_-transformed expression values; all preprocessing algorithms except MAS5 and MBEI return such values by default. RT-PCR log_2_ expression values are given as the negative of the C_T_ (threshold cycle). All results can be reproduced using the scripts provided in Text S1.

## Supporting Information

Dataset S1 A tab-delimited text file containing raw RT-PCR CT values for the cell line data set.
(0.03 MB TXT)

Dataset S2 A tab-delimited text file containing raw RT-PCR CT values for the colon data set.
(0.03 MB TXT)

Table S1 An Excel spreadsheet listing the comparable RT-PCR probes and microarray probe sets used in this study.
(0.03 MB XLS)

Text S1 A PDF document providing R code to reproduce the analysis described in the manuscript.
(0.32 MB PDF)
