# Supplementary material for: Evaluation of Microarray Preprocessing Algorithms Based on Concordance with RT-PCR in Clinical Samples
Source: PLoS One. 2009 May 21;4(5):e5645. doi: 10.1371/journal.pone.0005645 (PMC2680989; doi:10.1371/journal.pone.0005645)
Supplement: Text S1 — A PDF document providing R code to reproduce the analysis described in the manuscript. (0.32 MB PDF) [file pone.0005645.s004.pdf]

# Supplemental Material: Choice of normalization algorithm affects consistency between microarray and RT-PCR in clinical samples

Balazs Gyorffy  Bela Molnar  Hermann Lage  Zoltan Szallasi
Aron C. Eklund

June 27, 2008

The following R code downloads the microarray data from GEO and processes it to create the AffyBatch objects.

## 1 Getting started

```
> library(affy)
```

The following functions are hacks based on an older version of the GEOquery package:

```
> parseGeoMeta <- function(txt) {
+     leader <- strsplit(grep("!\\w*_", txt, perl = TRUE, value = TRUE)[1],
+         "_")[[1]][1]
+     tmp <- txt[grep(leader, txt)]
+     tmp <- gsub(paste(leader, "_", sep = ""), "", tmp)
+     first.eq <- regexpr(" = ", tmp)
+     tmp <- cbind(substring(tmp, first = 1, last = first.eq -
+         1), substring(tmp, first = first.eq + 3))
+     header <- split(tmp[, 2], tmp[, 1])
+     return(header)
+ }
> getMetaGEO <- function(GEOAccNum, drop = TRUE) {
+     pre <- "http://www.ncbi.nlm.nih.gov/geo/query/acc.cgi?acc="
+     post <- "&targ=gsm&form=text&view=brief"
+     txt <- readLines(paste(pre, GEOAccNum, post, sep = ""))
+     meta <- parseGeoMeta(txt)
+     lng <- sapply(meta, length)
+     meta2 <- meta[lng == median(lng)]
+     if (drop) {
+         n.levels <- sapply(meta2, function(x) length(levels(factor(x))))
+         meta2 <- meta2[n.levels > 1]
+     }
+     as.data.frame(meta2)
+ }
```

## 2 GSE11812: cell lines

```
> system(paste("curl -O ", "ftp://ftp.ncbi.nih.gov/pub/geo/DATA/supplementary/",
+     "series/GSE11812/GSE11812_RAW.tar", sep = ""))
> system("tar xf GSE11812_RAW.tar")

> gse11812.meta <- getMetaGEO("GSE11812", drop = TRUE)
> rownames(gse11812.meta) <- paste(gse11812.meta$geo_accession,
+     ".CEL.gz", sep = "")
> all(rownames(gse11812.meta) %in% dir())

[1] TRUE

> gse11812.p <- gse11812.meta[, c(1, 2, 5)]
> gse11812.batch <- ReadAffy(filenames = rownames(gse11812.p),
+     phenoData = as(gse11812.p, "AnnotatedDataFrame"), notes = "GSE11812, cell lines from
> save(gse11812.batch, file = "gse11812.batch.RData")
> gse11812.batch

AffyBatch object
size of arrays=712x712 features (24 kb)
cdf=HG-U133A (22283 affyids)
number of samples=30
number of genes=22283
annotation=hgu133a
notes= GSE11812, cell lines from B. Gyorffy et al
```

## 3 GSE4183: colon biopsies

```
> system(paste("curl -O ", "ftp://ftp.ncbi.nih.gov/pub/geo/DATA/supplementary/",
+     "series/GSE4183/GSE4183_RAW.tar", sep = ""))
> system("tar xf GSE4183_RAW.tar")

> gse4183.meta <- getMetaGEO("GSE4183", drop = TRUE)
> rownames(gse4183.meta) <- paste(gse4183.meta$geo_accession, ".CEL.gz",
+     sep = "")
> all(rownames(gse4183.meta) %in% dir())

[1] TRUE

> gse4183.p <- gse4183.meta[, c(1, 2, 4)]
> gse4183.batch <- ReadAffy(filenames = rownames(gse4183.p), phenoData = as(gse4183.p,
+     "AnnotatedDataFrame"), notes = "GSE4183, colon biopsies from B. Gyorffy et al")
> save(gse4183.batch, file = "gse4183.batch.RData")
> gse4183.batch

AffyBatch object
size of arrays=1164x1164 features (29 kb)
```

```
cdf=HG-U133_Plus_2 (54675 affyids)
number of samples=53
number of genes=54675
annotation=hgu133plus2
notes= GSE4183, colon biopsies from B. Gyorffy et al
```

# 4 sessionInfo

The results in this file are generated using the following packages:

```
> sessionInfo()

R version 2.6.1 (2007-11-26)
ia64-unknown-linux-gnu

locale:
LC_CTYPE=en_US.UTF-8;LC_NUMERIC=C;LC_TIME=en_US.UTF-8;LC_COLLATE=en_US.UTF-8;LC_MONETARY=e

attached base packages:
[1] tools     stats     graphics  grDevices utils     datasets  methods
[8] base

other attached packages:
[1] hgu133plus2cdf_2.0.0 hgu133acdf_2.0.0     affy_1.16.0
[4] preprocessCore_1.0.0 affyio_1.6.1         Biobase_1.16.3
```

# Supplemental Material: Choice of normalization algorithm affects consistency between microarray and RT-PCR in clinical samples

Balazs Gyorffy Bela Molnar Hermann Lage Zoltan Szallasi
Aron C. Eklund

June 29, 2008

The following R code processes the raw microarray probes intensities to generate expression values, using various normalization algorithms.

## 1 Getting started

```
> library(affy)
> library(farms)
> library(gcrma)
> library(plier)
> library(bgx)
> library(vsn)
```

Define some functions for those algorithms that have not been implemented as a simple R function.

```
> mbei <- function(x) expresso(x, normalize.method = "invariantset",
+     bg.correct = FALSE, pmcorrect.method = "pmonly", summary.method = "liwong")
> plierPlus16 <- function(x) {
+     e <- justPlier(x, normalize = TRUE)
+     exprs(e) <- log2((2^exprs(e)) + 16)
+     e
+ }
> source("dfwcode.R")
> dfw <- function(x) {
+     expresso(x, bgcorrect.method = "none", normalize.method = "quantiles",
+         pmcorrect.method = "pmonly", summary.method = "dfw")
+ }
```

Since it may be interesting to compare computation times for the various normalization algorithms, we will try to make the memory situations similar for each algorithm. Thus, we will perform garbage collection beforehand, and remove the results from memory afterwards.

```
> timeNormalize <- function(abatch, fn, name) {
+     gc(verbose = FALSE)
+     out <- system.time(assign(name, fn(abatch)))
+     save(name, file = paste(name, ".RData", sep = ""))
+     rm(name)
+     out
+ }
```

## 2 GSE11812: cell lines

### 2.1 raw data preparation

Load the raw data as available on the GEO site.

```
> load("gse11812.batch.RData")
```

Retain only those samples for which we have RT-PCR data.

```
> cells.pcr <- as.matrix(read.delim("Supplemental table 1_cell line rtpcr raw data.txt",
+     row.names = 1))
> dim(cells.pcr)

[1] 96 29

> dim(exprs(gse11812.batch))

[1] 506944     30

> cells.batch <- gse11812.batch[, match(colnames(cells.pcr), gse11812.batch$geo)]
> all(cells.batch$geo == colnames(cells.pcr))

[1] TRUE
```

Check for low-quality samples (based on the fraction of present calls for each sample).

```
> cells.mas5calls <- mas5calls(cells.batch)

Getting probe level data...
Computing p-values
Making P/M/A Calls

> cells.present <- colMeans(exprs(cells.mas5calls) == "P")

> plot(cells.present, ylab = "fraction present calls", main = "cell lines")
```

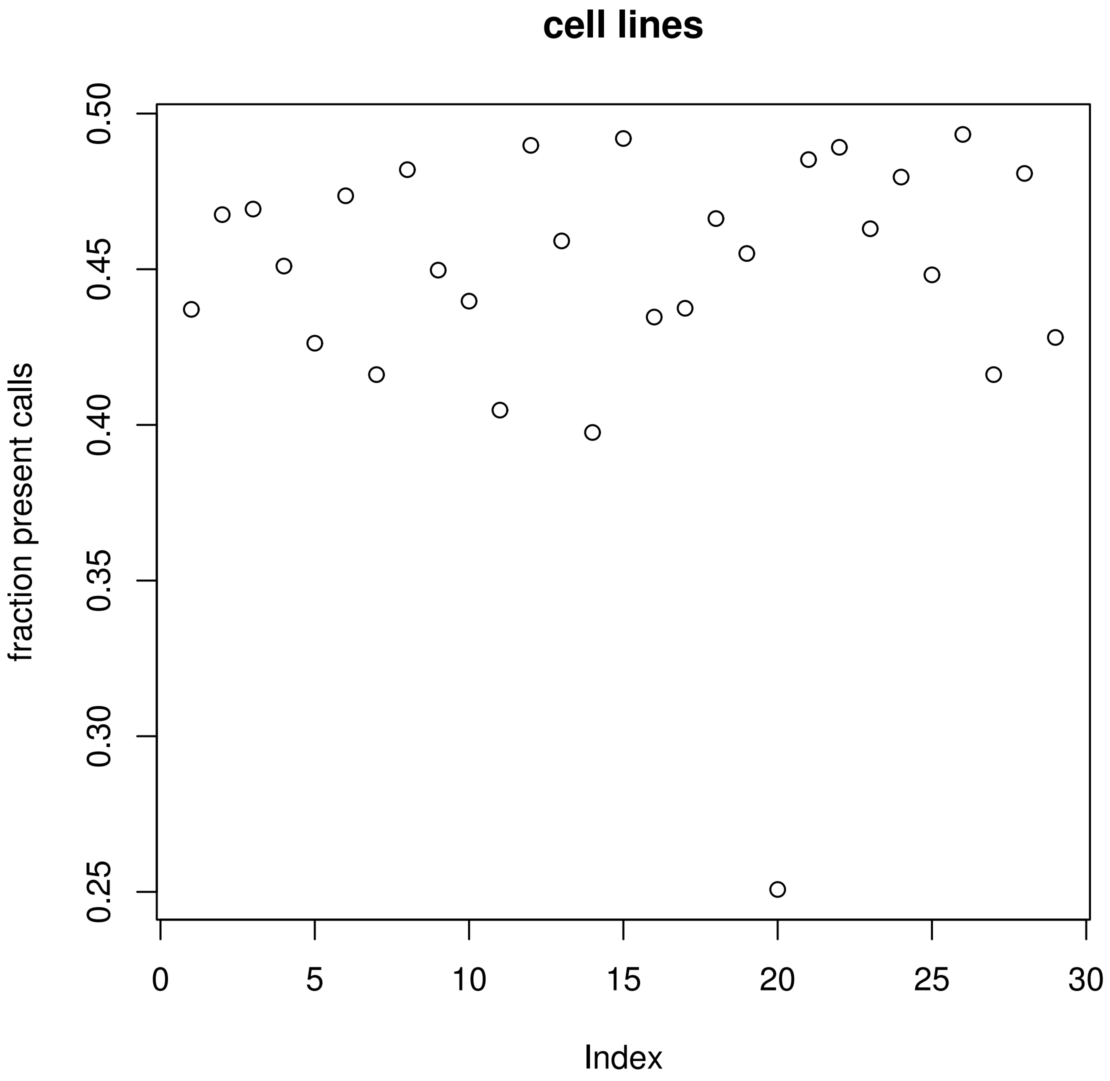


Sample #20 is a clear outlier, so remove it.

```
> cells.batch <- cells.batch[, -20]
> dim(exprs(cells.batch))

[1] 506944     28

> save(cells.batch, file = "cells.batch.RData")
```

"cells.batch" is our working raw data set.
Remove clutter.

```
> rm(gse11812.batch, cells.mas5calls)
```

## 2.2 normalization

Now process the raw data with the various normalization algorithms.

```
> timeNormalize(cells.batch, mas5, "cells.mas5")

background correction: mas
PM/MM correction : mas
expression values: mas
background correcting...done.
22283 ids to be processed
|                    |
|####################|
    user   system  elapsed
1974.750    0.776 1976.244

> timeNormalize(cells.batch, rma, "cells.rma")

Background correcting
Normalizing
Calculating Expression
   user  system elapsed
 62.791   1.667  64.484

> timeNormalize(cells.batch, gcrma, "cells.gcrma")

Adjusting for optical effect............................Done.
Computing affinities.Done.
Adjusting for non-specific binding...........................Done.
Normalizing
Calculating Expression
   user  system elapsed
519.584   8.661 529.349

> timeNormalize(cells.batch, mbei, "cells.mbei")

normalization: invariantset
PM/MM correction : pmonly
expression values: liwong
normalizing...done.
22283 ids to be processed
|                    |
|####################|
    user   system  elapsed
1577.821   15.627 1594.185

> timeNormalize(cells.batch, justPlier, "cells.plier")

   user  system elapsed
260.726   0.378 261.582
```

```
> timeNormalize(cells.batch, plierPlus16, "cells.plierPlus16")

Quantile normalizing...Done.
   user  system elapsed
317.979   0.486 318.801

> timeNormalize(cells.batch, q.farms, "cells.qfarms")

background correction: none
normalization: quantiles
PM/MM correction : pmonly
expression values: farms
background correcting...done.
normalizing...done.
22283 ids to be processed
|                    |
|####################|
   user  system elapsed
554.136   0.405 555.142

> timeNormalize(cells.batch, dfw, "cells.dfw")

background correction: none
normalization: quantiles
PM/MM correction : pmonly
expression values: dfw
background correcting...done.
normalizing...done.
22283 ids to be processed
|                    |
|####################|
   user  system elapsed
398.555   0.551 399.755

> timeNormalize(cells.batch, vsnrma, "cells.vsn")

Calculating Expression
   user  system elapsed
 77.587   0.713  78.456
```

# 3 GSE4183: colon biopsies

## 3.1 raw data preparation

```
> load("gse4183.batch.RData")
```

Retain only those samples for which we have RT-PCR data.

```
> colon.pcr <- as.matrix(read.delim("Supplemental table 2_colon rtpcr raw data.txt",
+     row.names = 1))
> dim(colon.pcr)
```

```
[1] 96 36
```

```
> dim(exprs(gse4183.batch))
```

```
[1] 1354896      53
```

```
> colon.batch <- gse4183.batch[, match(colnames(colon.pcr), gse4183.batch$geo)]
> all(colon.batch$geo == colnames(colon.pcr))
```

```
[1] TRUE
```

Check for low-quality samples (based on the fraction of present calls for each sample).

```
> colon.mas5calls <- mas5calls(colon.batch)
```

```
Getting probe level data...
Computing p-values
Making P/M/A Calls
```

```
> colon.present <- colMeans(exprs(colon.mas5calls) == "P")
```

```
> plot(colon.present, ylab = "fraction present calls", main = "colon biopsies")
```

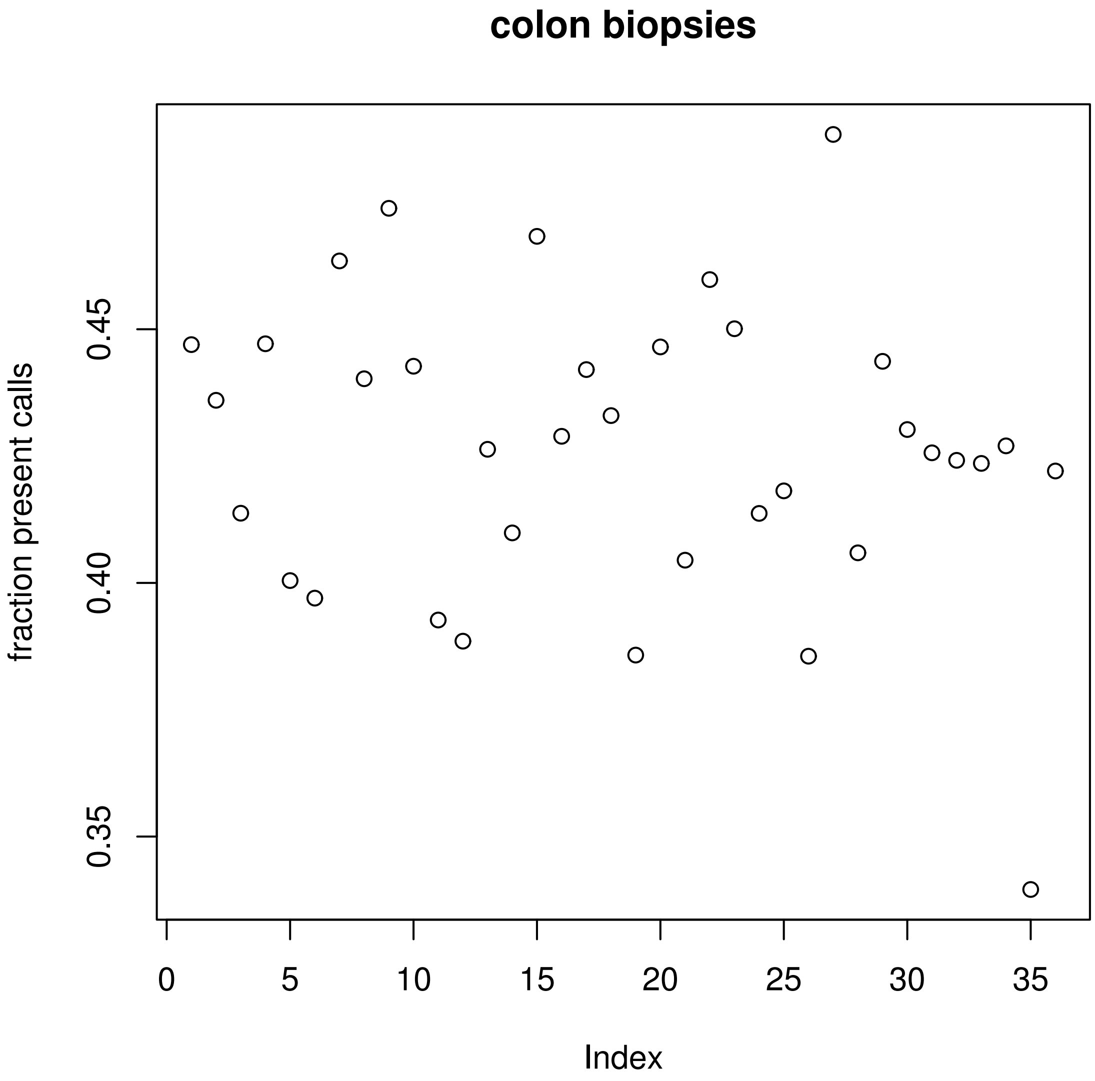


Perhaps sample #35 is kind of borderline, but we'll leave it in.

```
> dim(exprs(colon.batch))
```

```
[1] 1354896      36
```

```
> save(colon.batch, file = "colon.batch.RData")
```

"colon.batch" is our working raw data set.
Remove clutter.

```
> rm(gse4183.batch, colon.mas5calls)
```

### 3.2 normalization

Now process the raw data with the various normalization algorithms.

```
> timeNormalize(colon.batch, mas5, "colon.mas5")

background correction: mas
PM/MM correction : mas
expression values: mas
background correcting...done.
54675 ids to be processed
|                    |
|####################|
    user   system  elapsed
6431.496    2.375 6436.636

> timeNormalize(colon.batch, rma, "colon.rma")

Background correcting
Normalizing
Calculating Expression
   user  system elapsed
177.630   4.882 182.736

> timeNormalize(colon.batch, gcrma, "colon.gcrma")

Adjusting for optical effect...................................Done.
Computing affinities.Done.
Adjusting for non-specific binding...................................Done.
Normalizing
Calculating Expression
    user   system  elapsed
1874.221   28.706 1904.747

> timeNormalize(colon.batch, mbei, "colon.mbei")

normalization: invariantset
PM/MM correction : pmonly
expression values: liwong
normalizing...done.
54675 ids to be processed
|                    |
|####################|
    user   system  elapsed
5201.858   63.830 5267.499

> timeNormalize(colon.batch, justPlier, "colon.plier")

   user  system elapsed
837.825   0.772 838.665

> timeNormalize(colon.batch, plierPlus16, "colon.plierPlus16")
```

```
Quantile normalizing...Done.
    user   system  elapsed 
1001.807    2.235 1004.080 
```

```
> timeNormalize(colon.batch, q.farms, "colon.qfarms")
```

```
background correction: none 
normalization: quantiles 
PM/MM correction : pmonly 
expression values: farms 
background correcting...done.
normalizing...done.
54675 ids to be processed
|                    |
|####################|
    user   system  elapsed 
1231.954    1.993 1235.199 
```

```
> timeNormalize(colon.batch, dfw, "colon.dfw")
```

```
background correction: none 
normalization: quantiles 
PM/MM correction : pmonly 
expression values: dfw 
background correcting...done.
normalizing...done.
54675 ids to be processed
|                    |
|####################|
   user  system elapsed 
992.720   1.843 995.737 
```

```
> timeNormalize(colon.batch, vsnrma, "colon.vsn")
```

```
Calculating Expression
   user  system elapsed 
280.609   2.270 282.906 
```

## 4 sessionInfo

The results in this file are generated using the following packages:

```
> sessionInfo()
```

```
R version 2.6.1 (2007-11-26) 
ia64-unknown-linux-gnu 

locale:
```

```
LC_CTYPE=en_US.UTF-8;LC_NUMERIC=C;LC_TIME=en_US.UTF-8;LC_COLLATE=en_US.UTF-8;LC_MONETARY=e

attached base packages:
[1] splines   tools     stats     graphics  grDevices utils     datasets
[8] methods   base

other attached packages:
 [1] hgu133plus2probe_2.0.0 hgu133plus2cdf_2.0.0   hgu133aprobe_2.0.0
 [4] hgu133acdf_2.0.0       vsn_3.2.1              limma_2.12.0
 [7] bgx_1.2.2              plier_1.8.0            gcrma_2.10.0
[10] matchprobes_1.10.0     farms_1.3              MASS_7.2-38
[13] affy_1.16.0            preprocessCore_1.0.0   affyio_1.6.1
[16] Biobase_1.16.3

loaded via a namespace (and not attached):
[1] grid_2.6.1     lattice_0.17-2

> system("uname -a", intern = TRUE)

[1] "Linux sbiology 2.6.5-7.282-sn2 #1 SMP Tue Aug 29 10:40:40 UTC 2006 ia64 ia64 ia64 GNU
```

Supplemental Material: Choice of normalization algorithm affects consistency between microarray and RT-PCR in clinical samples

Balazs Gyorffy Bela Molnar Hermann Lage Zoltan Szallasi
Aron C. Eklund

June 30, 2008

This R script generates Figure 1, which displays the distribution of three bias metrics in various microarray data sets.

## 1 Calculate bias metrics

Load raw data and calculate bias metrics for each data set.

```
> library(affy)
> datasets <- unlist(list(LatinSquare133 = "SpikeIn133", LatinSquare95 = "SpikeIn95",
+     Hess_breast = "hess", Wang_breast = "wang", Sotiriou_breast = "sotiriou",
+     Pawitan_breast = "gse1456.u133a", Bild_lung = "bild.lung",
+     Gyorffy_colon = "colon", Gyorffy_cells = "cells"))
> getMetrics <- function(x) {
+     data.frame(degradation = AffyRNAdeg(x)$slope, pm.median = apply(log2(pm(x)),
+         2, median), present = colMeans(exprs(mas5calls(x)) ==
+         "P"))
+ }
> allMetrics <- lapply(datasets, function(x) {
+     batchname <- paste(x, ".batch", sep = "")
+     load(paste(batchname, ".RData", sep = ""))
+     m <- getMetrics(get(batchname))
+     rm(list = batchname)
+     m
+ })
> save(allMetrics, file = "allMetrics.RData")
```

Here is a quick summary of the data sets: number of samples, and the standard deviation of each bias metric:

```
> data.frame(n = sapply(allMetrics, nrow), t(sapply(allMetrics,
+     apply, 2, sd)))
```

```
                  n degradation pm.median    present
LatinSquare133   42  0.04856287 0.1288504 0.01241301
LatinSquare95    59  0.04902640 0.1423922 0.02044941
Hess_breast     133  0.59908528 0.6336858 0.06537147
Wang_breast     286  1.22407545 0.3191447 0.03899426
Sotiriou_breast 189  0.61529253 0.6374836 0.03282777
Pawitan_breast  159  0.88055092 0.3874275 0.04037465
Bild_lung       111  0.98951944 0.3067100 0.04298324
Gyorffy_colon    36  0.47342339 0.3467839 0.02921767
Gyorffy_cells    28  0.67439105 0.2501737 0.02775232
```

## 2 Draw Figure 1

```
> all.degradation <- lapply(allMetrics, function(x) x$degradation)
> all.pm.median <- lapply(allMetrics, function(x) x$pm.median)
> all.present <- lapply(allMetrics, function(x) x$present)
```

```
> par(mar = c(5, 1, 2, 1) + 0.1, oma = c(0, 7, 0, 1), las = 1,
+     mfrow = c(1, 3))
> boxplot(all.degradation, horizontal = T, names = names(datasets),
+     xlab = "a. RNA degradation slope")
> boxplot(all.pm.median, horizontal = T, names = NA, xlab = "b. median log2 PM intensity")
> boxplot(all.present, horizontal = T, names = NA, xlab = "c. fraction present calls")
```

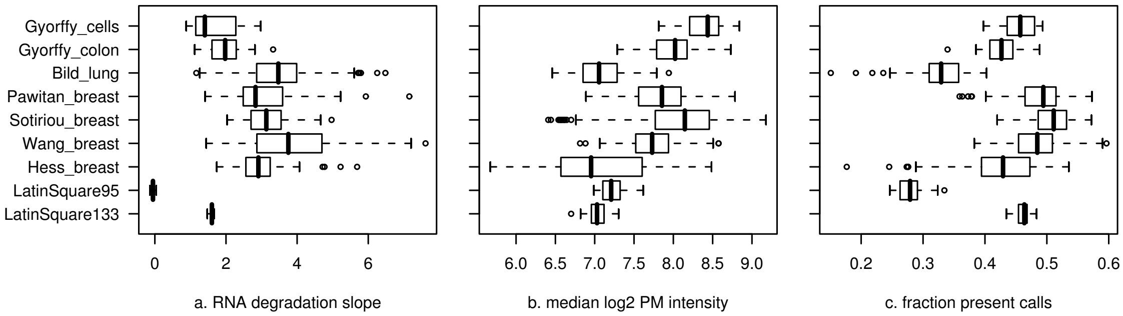


## 3 sessionInfo

The results in this file are generated using the following packages:

```
> sessionInfo()
```

```
R version 2.6.1 (2007-11-26)
ia64-unknown-linux-gnu

locale:
LC_CTYPE=en_US.UTF-8;LC_NUMERIC=C;LC_TIME=en_US.UTF-8;LC_COLLATE=en_US.UTF-8;LC_MONETARY=e
```

```
attached base packages:
[1] stats     graphics  grDevices utils     datasets  methods   base

> system("uname -a", intern = TRUE)

[1] "Linux sbiology 2.6.5-7.282-sn2 #1 SMP Tue Aug 29 10:40:40 UTC 2006 ia64 ia64 ia64 GNU
```

# Supplemental Material: Selecting a microarray preprocessing algorithm with highest concordance to RT-PCR in clinical samples

Balazs Gyorffy   Bela Molnar   Hermann Lage   Zoltan Szallasi
Aron C. Eklund

February 18, 2009

## 1 Collect all comparable data into one place.

```
> library(affy)
> library(gdata)
```

### 1.1 Load the probe lookup tables

The lookup files list probes that are comparable between microarray and RT-PCR.

```
> colon.lookup <- read.xls("SuppFile1.xls", sheet = 2, stringsAsFactors = FALSE)

Converting xls file to csv file... Done.
Reading csv file... Done.

> cells.lookup <- read.xls("SuppFile1.xls", sheet = 1, stringsAsFactors = FALSE)

Converting xls file to csv file... Done.
Reading csv file... Done.
```

### 1.2 Load the raw RT-PCR data (CT values)

```
> colon.rtpcr <- as.matrix(read.delim("Supplemental table 2_colon rtpcr raw data.txt",
+     row.names = 1))
> cells.rtpcr <- as.matrix(read.delim("Supplemental table 1_cell line rtpcr raw data.txt",
+     row.names = 1))
```

#### 1.2.1 Remove the RT-PCR sample corresponding to the bad microarray sample

```
> cells.rtpcr <- cells.rtpcr[, -20]
```

### 1.3 Confirm that the sample names are consistent between microarray and RT-PCR

```
> load("colon.rma.RData")
> load("cells.rma.RData")
> all(colon.rma$geo_accession == colnames(colon.rtpcr))
```

```
[1] TRUE
```

```
> all(cells.rma$geo_accession == colnames(cells.rtpcr))
```

```
[1] TRUE
```

### 1.4 Define the RT-PCR normalization algorithm

The general scheme is to normalize the values from each sample to the 18S rRNA probe.

The raw data is given as $C_T$ values, which are proportional to the negative of the $\log_2$ expression value.

For aesthetic reasons, we will scale the RT-PCR values such that all $\log_2$ expression values are positive.

```
> range(cells.rtpcr, colon.rtpcr, na.rm = TRUE)
```

```
[1]  7.515985 39.976204
```

Thus, we will arbitrarily set the 18S value to 40.

The following function normalizes RT-PCR data to 18S rRNA.

```
> norm.18s <- function(x) {
+     40 - sweep(x, 2, x["4342379-18S", ])
+ }
```

### 1.5 Combine data

Combine RT-PCR expression values and microarray expression values (from several normalization algorithms). Only the probes comparable across platforms are retained.

```
> colon2 <- list(rtpcr = norm.18s(colon.rtpcr)[colon.lookup$taqman.probe,
+     ])
> cells2 <- list(rtpcr = norm.18s(cells.rtpcr)[cells.lookup$taqman.probe,
+     ])
> algorithms <- unlist(list(RMA = "rma", MAS5 = "mas5", GCRMA = "gcrma",
+     MBEI = "mbei", PLIER = "plier", "PLIER+16" = "plierPlus16",
+     DFW = "dfw", FARMS = "qfarms", VSN = "vsn"))
> for (i in algorithms) {
+     objectName <- paste("cells.", i, sep = "")
+     filename <- paste(objectName, ".RData", sep = "")
+     load(filename)
+     cells2[[i]] <- exprs(get(objectName))[cells.lookup$affymetrix.probe,
+         ]
```

```
+     rm(list = objectName)
+ }
> for (i in algorithms) {
+     objectName <- paste("colon.", i, sep = "")
+     filename <- paste(objectName, ".RData", sep = "")
+     load(filename)
+     colon2[[i]] <- exprs(get(objectName))[colon.lookup$affymetrix.probe,
+         ]
+     rm(list = objectName)
+ }
```

### 1.6 $\log_2$-transform the MAS5 and MBEI expression values

This makes MAS5 and MBEI expression values comparable with the values from the other algorithms, which are already $\log_2$-transformed.

```
> cells2$mas5 <- log2(cells2$mas5)
> colon2$mas5 <- log2(colon2$mas5)
> cells2$mbei <- log2(cells2$mbei)
> colon2$mbei <- log2(colon2$mbei)
```

### 1.7 An overview of the data objects

```
> str(cells2)
```

```
List of 10
 $ rtpcr      : num [1:75, 1:28] 27.9 14.4 17.3 18.0 23.1 ...
  ..- attr(*, "dimnames")=List of 2
  .. ..$ : chr [1:75] "Hs00153340_m1" "Hs00154079_m1" "Hs00155308_m1" "Hs00156076_m1" ...
  .. ..$ : chr [1:28] "GSM298625" "GSM298626" "GSM298627" "GSM298628" ...
 $ rma        : num [1:75, 1:28] 7.52 7.12 7.38 7.06 5.09 ...
  ..- attr(*, "dimnames")=List of 2
  .. ..$ : chr [1:75] "211300_s_at" "205082_s_at" "202967_at" "201261_x_at" ...
  .. ..$ : chr [1:28] "GSM298625.CEL.gz" "GSM298626.CEL.gz" "GSM298627.CEL.gz" "GSM298628.
 $ mas5       : num [1:75, 1:28] 8.01 3.85 7.84 7.98 7.27 ...
  ..- attr(*, "dimnames")=List of 2
  .. ..$ : chr [1:75] "211300_s_at" "205082_s_at" "202967_at" "201261_x_at" ...
  .. ..$ : chr [1:28] "GSM298625.CEL.gz" "GSM298626.CEL.gz" "GSM298627.CEL.gz" "GSM298628.
 $ gcrma      : num [1:75, 1:28] 7.40 2.59 4.23 2.62 6.94 ...
  ..- attr(*, "dimnames")=List of 2
  .. ..$ : chr [1:75] "211300_s_at" "205082_s_at" "202967_at" "201261_x_at" ...
  .. ..$ : chr [1:28] "GSM298625.CEL.gz" "GSM298626.CEL.gz" "GSM298627.CEL.gz" "GSM298628.
 $ mbei       : num [1:75, 1:28] 8.71 8.67 8.61 8.19 9.19 ...
  ..- attr(*, "dimnames")=List of 2
  .. ..$ : chr [1:75] "211300_s_at" "205082_s_at" "202967_at" "201261_x_at" ...
  .. ..$ : chr [1:28] "GSM298625.CEL.gz" "GSM298626.CEL.gz" "GSM298627.CEL.gz" "GSM298628.
 $ plier      : num [1:75, 1:28] 6.58 5.37 5.31 6.46 8.07 ...
  ..- attr(*, "dimnames")=List of 2
```

```
 .. ..$ : chr [1:75] "211300_s_at" "205082_s_at" "202967_at" "201261_x_at" ...
 .. ..$ : chr [1:28] "GSM298625.CEL.gz" "GSM298626.CEL.gz" "GSM298627.CEL.gz" "GSM298628.
 $ plierPlus16: num [1:75, 1:28] 7.04 6.02 6.25 6.94 8.39 ...
  ..- attr(*, "dimnames")=List of 2
  .. ..$ : chr [1:75] "211300_s_at" "205082_s_at" "202967_at" "201261_x_at" ...
  .. ..$ : chr [1:28] "GSM298625.CEL.gz" "GSM298626.CEL.gz" "GSM298627.CEL.gz" "GSM298628.
 $ dfw        : num [1:75, 1:28] 8.10 8.25 8.32 8.10 6.86 ...
  ..- attr(*, "dimnames")=List of 2
  .. ..$ : chr [1:75] "211300_s_at" "205082_s_at" "202967_at" "201261_x_at" ...
  .. ..$ : chr [1:28] "GSM298625.CEL.gz" "GSM298626.CEL.gz" "GSM298627.CEL.gz" "GSM298628.
 $ qfarms     : num [1:75, 1:28] 8.49 8.40 8.03 8.21 8.04 ...
  ..- attr(*, "dimnames")=List of 2
  .. ..$ : chr [1:75] "211300_s_at" "205082_s_at" "202967_at" "201261_x_at" ...
  .. ..$ : chr [1:28] "GSM298625.CEL.gz" "GSM298626.CEL.gz" "GSM298627.CEL.gz" "GSM298628.
 $ vsn        : num [1:75, 1:28] 8.35 8.15 8.29 8.18 7.36 ...
  ..- attr(*, "dimnames")=List of 2
  .. ..$ : chr [1:75] "211300_s_at" "205082_s_at" "202967_at" "201261_x_at" ...
  .. ..$ : chr [1:28] "GSM298625.CEL.gz" "GSM298626.CEL.gz" "GSM298627.CEL.gz" "GSM298628.
```

```
> str(colon2)
```

```
List of 10
 $ rtpcr      : num [1:84, 1:36] 18.9 18.5 23.6 22.8 23.7 ...
  ..- attr(*, "dimnames")=List of 2
  .. ..$ : chr [1:84] "Hs00152825_m1" "Hs00153133_m1" "Hs00153304_m1" "Hs00153350_m1" ...
  .. ..$ : chr [1:36] "GSM95525" "GSM95493" "GSM95494" "GSM95495" ...
 $ rma        : num [1:84, 1:36] 7.54 6.02 9.22 6.88 9.70 ...
  ..- attr(*, "dimnames")=List of 2
  .. ..$ : chr [1:84] "210166_at" "1554997_a_at" "204489_s_at" "203684_s_at" ...
  .. ..$ : chr [1:36] "GSM95525.CEL.gz" "GSM95493.CEL.gz" "GSM95494.CEL.gz" "GSM95495.CEL.
 $ mas5       : num [1:84, 1:36]  8.48  6.78 10.17  6.83 10.52 ...
  ..- attr(*, "dimnames")=List of 2
  .. ..$ : chr [1:84] "210166_at" "1554997_a_at" "204489_s_at" "203684_s_at" ...
  .. ..$ : chr [1:36] "GSM95525.CEL.gz" "GSM95493.CEL.gz" "GSM95494.CEL.gz" "GSM95495.CEL.
 $ gcrma      : num [1:84, 1:36] 3.74 2.67 9.45 2.80 9.75 ...
  ..- attr(*, "dimnames")=List of 2
  .. ..$ : chr [1:84] "210166_at" "1554997_a_at" "204489_s_at" "203684_s_at" ...
  .. ..$ : chr [1:36] "GSM95525.CEL.gz" "GSM95493.CEL.gz" "GSM95494.CEL.gz" "GSM95495.CEL.
 $ mbei       : num [1:84, 1:36]  8.35  7.59 11.10  8.93  9.80 ...
  ..- attr(*, "dimnames")=List of 2
  .. ..$ : chr [1:84] "210166_at" "1554997_a_at" "204489_s_at" "203684_s_at" ...
  .. ..$ : chr [1:36] "GSM95525.CEL.gz" "GSM95493.CEL.gz" "GSM95494.CEL.gz" "GSM95495.CEL.
 $ plier      : num [1:84, 1:36] 6.82 5.72 8.88 6.91 9.16 ...
  ..- attr(*, "dimnames")=List of 2
  .. ..$ : chr [1:84] "210166_at" "1554997_a_at" "204489_s_at" "203684_s_at" ...
  .. ..$ : chr [1:36] "GSM95525.CEL.gz" "GSM95493.CEL.gz" "GSM95494.CEL.gz" "GSM95495.CEL.
 $ plierPlus16: num [1:84, 1:36] 6.85 6.01 8.65 6.90 8.87 ...
```

```
  ..- attr(*, "dimnames")=List of 2
  .. ..$ : chr [1:84] "210166_at" "1554997_a_at" "204489_s_at" "203684_s_at" ...
  .. ..$ : chr [1:36] "GSM95525.CEL.gz" "GSM95493.CEL.gz" "GSM95494.CEL.gz" "GSM95495.CEL.
 $ dfw        : num [1:84, 1:36] 7.73 6.68 8.69 7.84 9.15 ...
  ..- attr(*, "dimnames")=List of 2
  .. ..$ : chr [1:84] "210166_at" "1554997_a_at" "204489_s_at" "203684_s_at" ...
  .. ..$ : chr [1:36] "GSM95525.CEL.gz" "GSM95493.CEL.gz" "GSM95494.CEL.gz" "GSM95495.CEL.
 $ qfarms     : num [1:84, 1:36] 8.17 6.88 8.91 7.93 8.74 ...
  ..- attr(*, "dimnames")=List of 2
  .. ..$ : chr [1:84] "210166_at" "1554997_a_at" "204489_s_at" "203684_s_at" ...
  .. ..$ : chr [1:36] "GSM95525.CEL.gz" "GSM95493.CEL.gz" "GSM95494.CEL.gz" "GSM95495.CEL.
 $ vsn        : num [1:84, 1:36] 7.98 7.15 9.45 7.48 9.86 ...
  ..- attr(*, "dimnames")=List of 2
  .. ..$ : chr [1:84] "210166_at" "1554997_a_at" "204489_s_at" "203684_s_at" ...
  .. ..$ : chr [1:36] "GSM95525.CEL.gz" "GSM95493.CEL.gz" "GSM95494.CEL.gz" "GSM95495.CEL.
```

### 1.8 Save the data objects for later use

```
> save(cells2, file = "cells2.RData")
> save(colon2, file = "colon2.RData")
```

## 2 sessionInfo

The results in this file are generated using the following packages:

```
> sessionInfo()

R version 2.7.1 (2008-06-23)
ia64-unknown-linux-gnu

locale:
C

attached base packages:
[1] tools     stats     graphics  grDevices utils     datasets  methods
[8] base

other attached packages:
[1] gdata_2.4.2          affy_1.18.2          preprocessCore_1.2.0
[4] affyio_1.8.0         Biobase_2.0.1

loaded via a namespace (and not attached):
[1] gtools_2.5.0

> system("uname -a", intern = TRUE)

[1] "Linux sbiology 2.6.16.54-0.2.5-default #1 SMP Mon Jan 21 13:29:51 UTC 2008 ia64 ia64
```

# Supplemental Material: Choice of normalization algorithm affects consistency between microarray and RT-PCR in clinical samples

Balazs Gyorffy Bela Molnar Hermann Lage Zoltan Szallasi
Aron C. Eklund

March 13, 2009

## 1 Evaluate concordance between RT-PCR and microarray using Pearson correlation and Log Ratio Discrepancy (LRD)

### 1.1 Load data

```
> load("colon2.RData")
> load("cells2.RData")
```

### 1.2 Normalize the data

Normalization function:

```
> normalize.median <- function(x) {
+     x.complete <- x[!apply(is.na(x), 1, any), ]
+     fac <- scale(apply(x.complete, 2, median), scale = FALSE)
+     sweep(x, 2, fac)
+ }
```

```
> colon2n <- lapply(colon2, normalize.median)
> cells2n <- lapply(cells2, normalize.median)
```

### 1.3 Define some functions

Define the function for log-ratio discrepancy:

```
> disc <- function(x, y, na.rm = FALSE, fn = mean) {
+     stopifnot(length(x) == length(y))
+     ok <- is.finite(x) & is.finite(y)
+     stopifnot(all(ok) || na.rm)
+     x <- x[ok]
+     y <- y[ok]
+     x.d <- outer(x, x, "-")
+     y.d <- outer(y, y, "-")
+     d <- (x.d - y.d)[upper.tri(x.d)]
```

```
+     fn(abs(d))
+ }
```

The algorithm names to use in the figures:

```
> algorithms <- c(RMA = "rma", MAS5 = "mas5", GCRMA = "gcrma",
+     MBEI = "mbei", PLIER = "plier", `PLIER+16` = "plierPlus16",
+     DFW = "dfw", FARMS = "qfarms", VSN = "vsn")
```

Apply a function to each gene, comparing RT-PCR to each microarray normalization algorithm:

```
> applyGene <- function(x, fn, ...) {
+     sapply(algorithms, function(y) {
+         res <- sapply(1:nrow(x[[y]]), function(i) fn(x[[y]][i,
+             ], x$rtpcr[i, ], ...))
+         names(res) <- rownames(x$rma)
+         res
+     })
+ }
```

Boxplot without whiskers or outliers

```
> boxplot2 <- function(x, col = NA, pars = list(), ylim, mean = TRUE,
+     horizontal = FALSE, ...) {
+     a <- boxplot(x, ..., plot = FALSE)
+     pars$boxfill <- col
+     if (missing(ylim))
+         ylim <- range(a$stats[2:4, ])
+     bxp(a, whisklty = 0, staplelty = 0, pars = pars, outline = FALSE,
+         ylim = ylim, horizontal = horizontal, ...)
+     invisible(a)
+ }
```

### 1.4 Pearson correlations

```
> colon.pcc <- applyGene(colon2n, cor, use = "pairwise")
> cells.pcc <- applyGene(cells2n, cor, use = "pairwise")
```

Define a convenient plotting function:

```
> p5 <- function(x, ...) {
+     d <- as.data.frame(x)
+     d <- d[, order(apply(d, 2, median, na.rm = TRUE))]
+     par(mar = c(5, 6, 2, 1) + 0.1, las = 1)
+     boxplot2(d, horizontal = TRUE, col = "lightgray", axes = FALSE,
+         xlab = "Pearson correlation", ...)
+     axis(1)
+     axis(2, at = 1:ncol(d), labels = colnames(d), tick = FALSE)
+ }
```

```
> par(mfrow = c(1, 2))
> p5(colon.pcc, main = "a. colon biopsies")
> p5(cells.pcc, main = "b. cell lines")
```

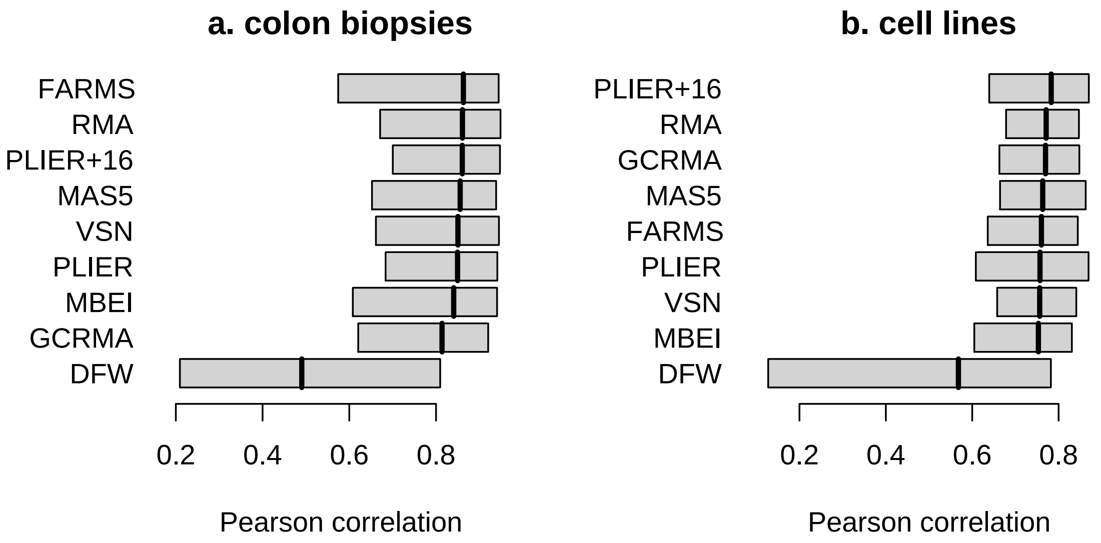

### 1.5 Log-ratio discrepancy (LRD)

```
> colon.lrd <- applyGene(colon2n, disc, na.rm = TRUE)
> cells.lrd <- applyGene(cells2n, disc, na.rm = TRUE)
```

Define a convenient plotting function:

```
> p6 <- function(x, ...) {
+     d <- as.data.frame(x)
+     d <- d[, order(apply(d, 2, median, na.rm = TRUE), decreasing = TRUE)]
+     par(mar = c(5, 6, 2, 1) + 0.1, las = 1)
+     boxplot2(d, horizontal = TRUE, col = "lightgray", axes = FALSE,
+         xlab = "log ratio discrepancy", ...)
+     axis(1)
+     axis(2, at = 1:ncol(d), labels = colnames(d), tick = FALSE)
+ }
```

```
> par(mfrow = c(1, 2))
> p6(colon.lrd, main = "a. colon biopsies")
> p6(cells.lrd, main = "b. cell lines")
```

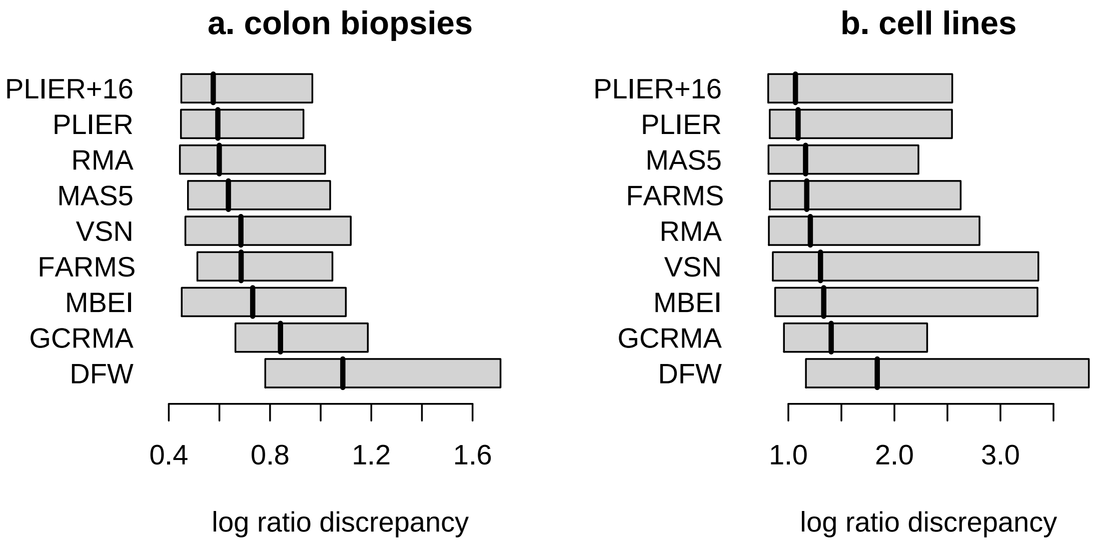

## 1.6 Is there a statistically significant difference between microarray normalization algorithms?

### 1.6.1 define some functions

We need my ”squash” package for the ”distogram” plot.

```
> library(squash)
```

”dist” for an arbitrary function

```
> afdist <- function(x, f, ...) {
+     x <- as.matrix(x)
+     n <- nrow(x)
+     k <- 1
+     out <- numeric(n * (n - 1)/2)
+     for (i in 1:(n - 1)) {
+         for (j in (i + 1):n) {
+             out[k] <- f(x[i, ], x[j, ], ...)
+             k <- k + 1
+         }
+     }
+     attr(out, "Size") <- n
+     attr(out, "Labels") <- rownames(x)
+     attr(out, "Diag") <- FALSE
+     attr(out, "Upper") <- FALSE
+     class(out) <- "dist"
+     out
+ }
```

This function creates a ”dist” object based on Wilcoxon P value.

```
> wilcox.dist <- function(x) {
+     wilcox.p <- function(x, y) wilcox.test(x, y)$p.value
+     afdist(x, wilcox.p)
+ }
```

Define the color scale for indicating P values.

```
> map <- colormap(breaks = c(0, 0.01, 0.05, 0.1, 1), colFn = function(n) c("red",
+     "orange", "yellow", "white"))
> names(map$colors) <- c("p < 0.01", "0.01 < p < 0.05", "0.05 < p < 0.1",
+     "0.1 < p < 1")
```

A function to sort by median, calculate wilcoxon P values, and plot.

```
> p7 <- function(x, decreasing = FALSE, ...) {
+     m <- apply(x, 2, median)
+     x <- x[, order(m, decreasing = decreasing)]
+     d <- wilcox.dist(t(x))
+     distogram(d, map = map, key = FALSE, ...)
+ }
```

### 1.6.2 Pearson correlation

```
> par(mfrow = c(1, 3), mar = c(2, 2, 4, 2))
> p7(colon.pcc, main = "a. colon biopsies")
> p7(cells.pcc, main = "b. cell lines")
> par(mar = c(2, 0, 4, 0))
> plot.new()
> legend.map("bottomleft", map = map, title = "Wilcoxon test",
+     bty = "n")
```

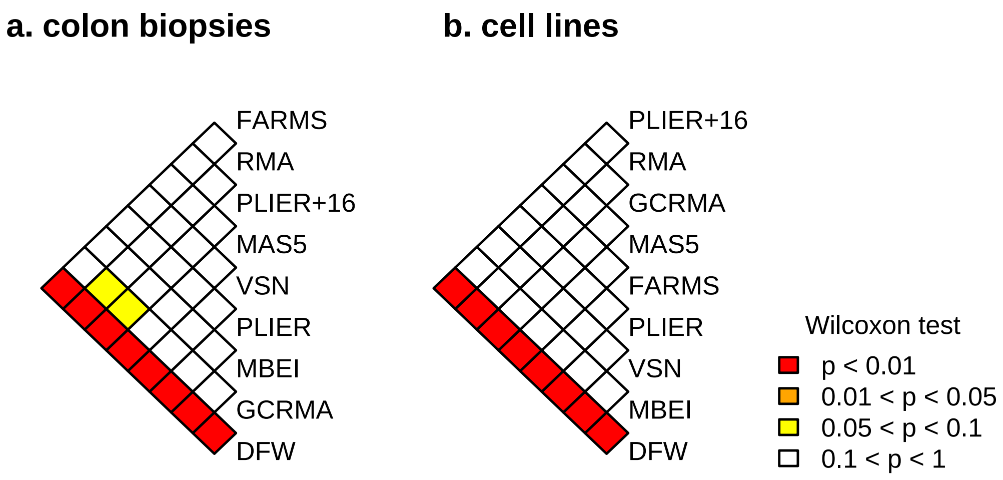

### 1.6.3 LRD

```
> par(mfrow = c(1, 3), mar = c(2, 2, 4, 2))
> p7(colon.lrd, TRUE, main = "a. colon biopsies")
> p7(cells.lrd, TRUE, main = "b. cell lines")
> par(mar = c(2, 0, 4, 0))
> plot.new()
> legend.map("bottomleft", map = map, title = "Wilcoxon test",
+     bty = "n")
```

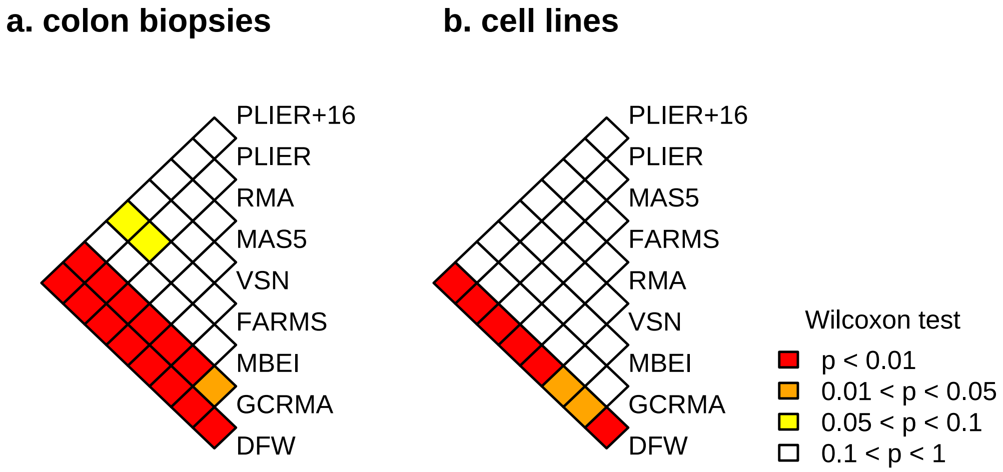

### 1.7 Combined figures

### 1.7.1 Pearson correlation

```
> layout(matrix(1:4, nrow = 2), widths = c(2, 1))
> par(mar = c(5, 6, 2, 1))
> p5(colon.pcc, main = "")
> mtext("a. colon biopsies", adj = 0, line = 0.5, font = 2)
> p5(cells.pcc, main = "")
> mtext("b. cell lines", adj = 0, line = 0.5, font = 2)
> par(mar = c(5, 1, 2, 1))
> p7(colon.pcc, main = "")
> p7(cells.pcc, main = "")
> par(fig = c(0, 1, 0, 1), usr = c(0, 1, 0, 1), xpd = NA, new = TRUE)
> legend.map("right", map = map, bty = "n", inset = -0.05)
```

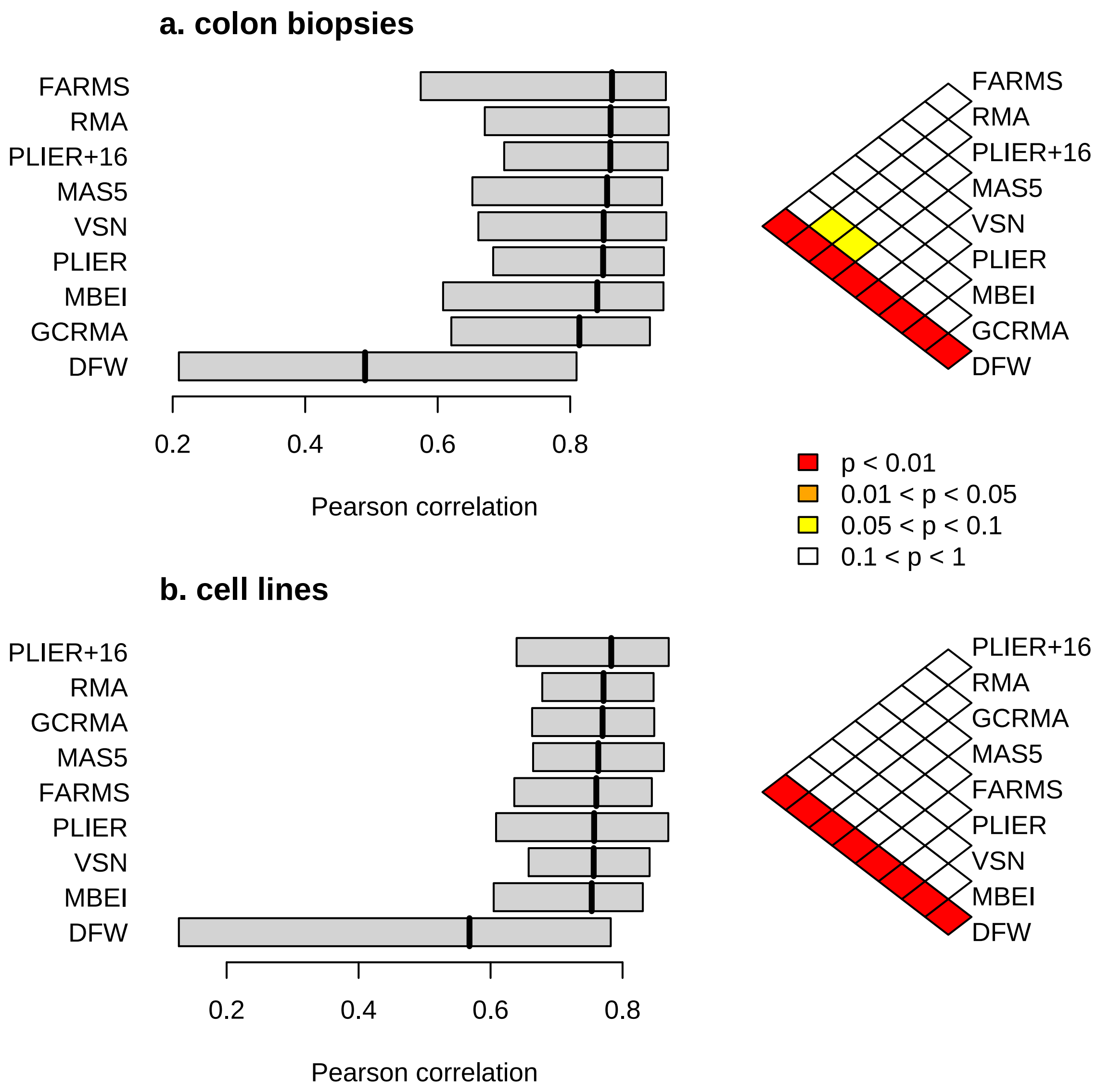

### 1.7.2 LRD

```
> layout(matrix(1:4, nrow = 2), widths = c(2, 1))
> par(mar = c(5, 6, 2, 1))
> p6(colon.lrd, main = "")
> mtext("a. colon biopsies", adj = 0, line = 0.5, font = 2)
> p6(cells.lrd, main = "")
> mtext("b. cell lines", adj = 0, line = 0.5, font = 2)
> par(mar = c(5, 1, 2, 1))
> p7(colon.lrd, TRUE, main = "")
> p7(cells.lrd, TRUE, main = "")
> par(fig = c(0, 1, 0, 1), usr = c(0, 1, 0, 1), new = TRUE)
> legend.map("right", map = map, bty = "n")
```

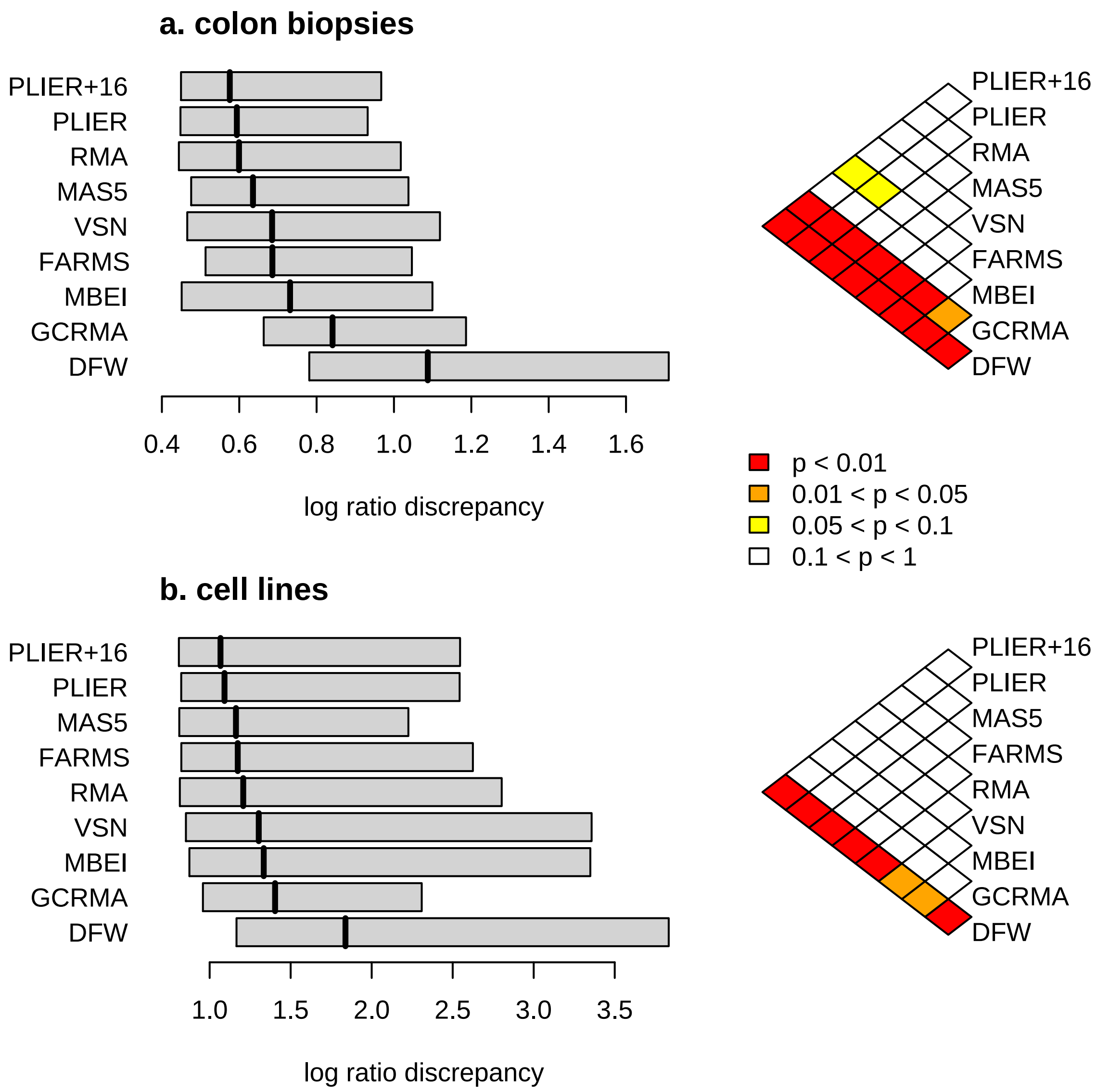

## 2 sessionInfo

The results in this file are generated using the following packages:

```
> sessionInfo()

R version 2.8.1 (2008-12-22)
i386-apple-darwin8.11.1

locale:
en_US.UTF-8/en_US.UTF-8/C/C/en_US.UTF-8/en_US.UTF-8

attached base packages:
[1] stats     graphics  grDevices utils     datasets  methods   base

other attached packages:
[1] squash_0.2.0

> system("uname -a", intern = TRUE)

[1] "Darwin mangle.local 9.6.0 Darwin Kernel Version 9.6.0: Mon Nov 24 17:37:00 PST 2008;
```
